# Expectations and Perceptions of Healthcare Professionals for Robot Deployment in Hospital Environments During the COVID-19 Pandemic

**DOI:** 10.3389/frobt.2021.612746

**Published:** 2021-06-02

**Authors:** Sergio D. Sierra Marín, Daniel Gomez-Vargas, Nathalia Céspedes, Marcela Múnera, Flavio Roberti, Patricio Barria, Subramanian Ramamoorthy, Marcelo Becker, Ricardo Carelli, Carlos A. Cifuentes

**Affiliations:** ^1^Department of Biomedical Engineering, Colombian School of Engineering Julio Garavito, Bogota, Colombia; ^2^Institute of Automatics, National University of San Juan, San Juan, Argentina; ^3^Club de Leones Cruz del Sur Rehabilitation Center, Punta Arenas, Chile; ^4^School of Informatics, University of Edinburgh, Edinburgh, United Kingdom; ^5^Department of Mechanical Engineering, São Carlos School of Engineering, University of São Paulo, São Carlos, Brazil

**Keywords:** robotics, healthcare professionals' expectations, COVID-19, hospital environments, robot applications, UV robot, telemedicine, survey

## Abstract

Several challenges to guarantee medical care have been exposed during the current COVID-19 pandemic. Although the literature has shown some robotics applications to overcome the potential hazards and risks in hospital environments, the implementation of those developments is limited, and few studies measure the perception and the acceptance of clinicians. This work presents the design and implementation of several perception questionnaires to assess healthcare provider's level of acceptance and education toward robotics for COVID-19 control in clinic scenarios. Specifically, 41 healthcare professionals satisfactorily accomplished the surveys, exhibiting a low level of knowledge about robotics applications in this scenario. Likewise, the surveys revealed that the fear of being replaced by robots remains in the medical community. In the Colombian context, 82.9% of participants indicated a positive perception concerning the development and implementation of robotics in clinic environments. Finally, in general terms, the participants exhibited a positive attitude toward using robots and recommended them to be used in the current panorama.

## 1. Introduction

The recent outbreak of COVID-19, caused by the new severe acute respiratory syndrome coronavirus 2 (SARS-CoV-2), has spread globally in an unprecedented way around the world (World Health Organization, 2020c). In the last months, the number of infections and deaths worldwide was alarming. Thus, the efforts of most countries were focused on containing and mitigating the effects of the pandemic (United Nations Development Programme, 2020; World Health Organization, 2020c). Given the transmission rate of the virus, the World Health Organization (WHO) recommended several strategies, such as physical distancing to prevent the transmission of COVID-19 (World Health Organization, 2020a). However, some countries are now resuming economic activities, and compliance with bio-safety protocols is still necessary to prevent the spread of the virus (Center for Disease Control and Prevention, 2020; Favero et al., 2020). In this context, to mitigate the effects of the COVID-19 pandemic, different public health measures have been adopted around the world with multiple impacts on the social, economic, and political sectors (Douglas et al., 2020; The World Bank, 2020; World Health Organization, 2020b).

Regarding the health sector, all levels and stakeholders of the world's health systems have been mainly committed to provide medical care during the pandemic (Barroy et al., 2020; Government of Canada, 2020; World Health Organization, 2020b). Hence, numerous challenges have arisen, such as (1) the vulnerability and overloading of healthcare professionals, (2) the decongestion and reduction of the risk of contagion in intra-hospital environments, (3) the availability of biomedical technology, and (3) the sustainability of patient care (Chatterjee and Kagwe, 2020; Government of Canada, 2020; Yang et al., 2020). Under this scenario, multiple strategies have been proposed to address such challenges. For instance, robotics that is a promising solution to help control and mitigate the effects of the COVID-19 pandemic (Boston Dynamics, 2020; EuRobotics, 2020; Javaid et al., 2020; Yang et al., 2020). Historically, robotics has assisted humans in a large number of fields, given its ability to execute tasks with precision, carry out industrial operations efficiently, interact in hostile environments, and execute highly complex works (Siciliano and Khatib, 2016; Cresswell et al., 2018; Nayak et al., 2020). Therefore, the applicability of robotics in society has been evident and is significantly growing (Siciliano and Khatib, 2016).

Overall, as multiple experts have discussed, robotics are potentially applicable in hospital environments during the pandemic for: (1) disinfection and sterilization of facilities, (2) handling and delivery of drugs, food, and waste, (3) telemedicine and remote assistance, as well as (4) detection and identification of new cases (Cresswell et al., 2018; Aymerich-Franch, 2020; Demaitre, 2020; Yang et al., 2020). For the first application, the implemented robot types commonly use ultraviolet (UV) lights, vaporization techniques, and vacuuming to guarantee disinfection or sterilization. This way, those devices show advantages in pathogen elimination and cleaning places, which could result in reduction of contagion risk (Yang et al., 2020).

Within the logistics and service context, robotic devices mainly apply mobile and aerial systems in delivery and supply production tasks (Yang et al., 2020). However, aerial robots could be unworkable for hospital environments. On the other hand, devices based on manipulators and hybrid systems (i.e., mobile base and manipulators) can also work in this application, focusing on these same tasks and supporting patient management (Yang et al., 2020). In telemedicine and telepresence applications, social robots and virtual agents are commonly implemented (Aymerich-Franch, 2020; Yang et al., 2020). Thus, these robotic systems allow providing benefits in aspects, such as accompanying, monitoring, and patrolling (Yang et al., 2020). Finally, for the detection and control applications, the motivation lies in monitoring of vital signs for clinical environments (Yang et al., 2020). Therefore, devices focused on this application covers hybrid mechanisms, aerial systems, or social robots.

Table 1 summarizes the most common types of robotic applications mentioned above applied to clinical environments and their potential benefits during the COVID-19 pandemic.

**Table 1:** Medical robotics applications that are potentially useful in combating the spread of COVID-19.

**Application**	**Robot type**	**Benefits**	**Suitable for hospitals?**	**Category in this study**
Disinfection, and cleaning	UV	Pathogen elimination.	Yes	DIS
	Vaporization	Reduction of the risk of contagion.	Yes	DIS
	Vacuuming	Cleaning	Yes	–
Logistics and service	Mobile	Waste and/or sample management. Delivery of food and medicines. Delivery of instrumentation.	Yes	ASL
	Aerial		No	–
	Manipulator	Supply production. Waste and/or sample management.	Yes	–
	Hybrid	Supply production. Waste and/or sample management. Delivery of food and medicine Delivery of instrumentation. Patient management	Yes	ASL
Telemedicine and telepresence	Virtual agents	Accompanying. Remote monitoring.	Yes	TEL
	Social	Accompanying. Remote monitoring.	Yes	TEL
	Hybrid	Patrolling and awareness.	Yes	TEL
Detection and control	Hybrid	Vital signs monitoring Patrolling and awareness	Yes	ASL
	Social		Yes	–
	Aerial		No	–

*TEL stands for Telemedicine, DIS stands for Disinfection and Cleaning, and ASL stands for Assistance, Service, and Logistics*.

Despite the above, only a few studies have been focused on measuring the perception and acceptance of healthcare providers toward robotic tools in the COVID-19 pandemic (Betriana et al., 2020; Miner et al., 2020; Viswanathan et al., 2020). Several studies analyzing the acceptability and adherence of technology in healthcare, such as home healthcare robots and information systems, have shown that more than 40% of these technologies have failed or have been abandoned in the last two decades (Alaiad and Zhou, 2014; Greenhalgh et al., 2017). One of the primary adoption barriers is an inadequate understanding of the socio-technical aspects of the technology, as well as users' knowledge and perception (Aarts, 2004). Due to this reason, different methods to measure attitudes and perceptions have been implemented (Krägeloh et al., 2019). Measuring such parameters, robot developers and engineering teams can understand the users' needs and expectations (Macdonald, 2009), as well as to have an initial insight into the usability of robot applications within the involved scenarios (Shinohara, 2012; Riek, 2017).

Accordingly, the current study aims to measure clinicians' knowledge and perception toward healthcare robotics for the COVID-19 pandemic. It is expected that a positive perception/attitude toward robotics and a high level of knowledge might promote better acceptability, adherence, and adoption of robots. Hence, a Knowledge, Attitudes, and Practices (KAP) questionnaire was developed. This questionnaire collects the data on the knowledge (i.e., what is known), attitudes (i.e., what is perceived), and practices (i.e., what is done) of a particular population (World Health Organization, 2014). In this case, 41 healthcare professionals (e.g., nurses, doctors, biomedical engineers, among others) participated in the study, assessing three categories: (DIS) Disinfection and cleaning robots, (ASL) Assistance, Service, and Logistics robots, and (TEL) Telemedicine and Telepresence robots 1.

The remainder of this work is organized as follows. Section 2 describes multiple robotic devices that have been reported in the literature to be useful for disinfection, assistance, and telemedicine. Section 3 outlines the experimental protocol and the perception questionnaires carried out in the study. Section 4 and 5 describes the primary outcomes of this study and the discussion. Finally, section 6 shows the main findings of this work.

## 2. Robotics for COVID-19 Pandemic

**Figure 1:**
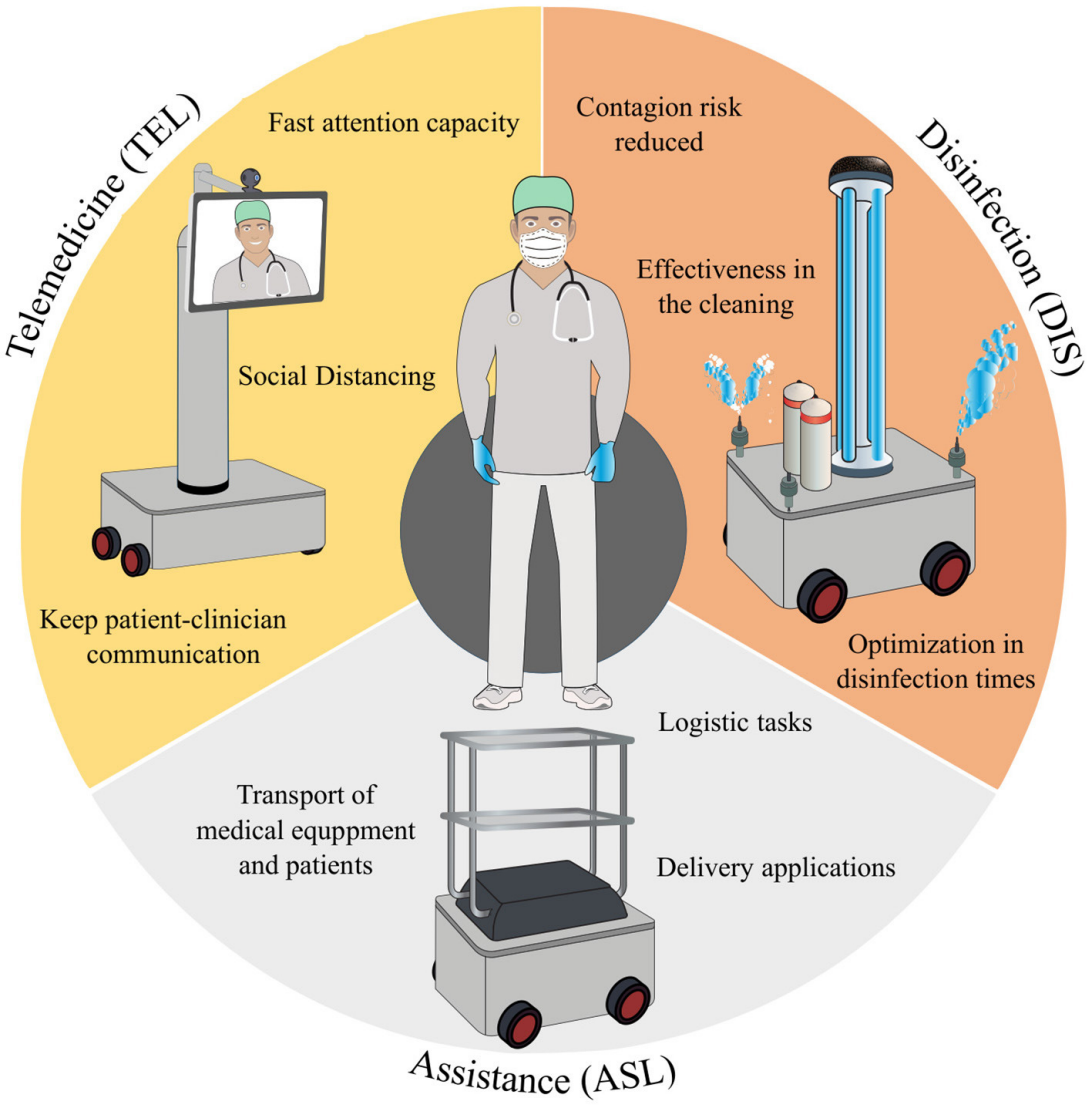
Categories of robotics application considered in this study: (1) Telemedicine (TEL), Disinfection (DIS), and Assistance (ASL). The exhibited characteristics refer to the main advantages of using robots of the corresponding category (i.e., the yellow section for TEL, orange for DIS, and gray for ASL) in clinic environments.

As described in Table 1, robotics for COVID-19 in hospital environments covers a wide range of possibilities. In this sense, multiple research groups worldwide have focused their efforts on developing strategies against the pandemic (Boston Dynamics, 2020; EuRobotics, 2020; Maxon Motors Inc., 2020; Robotnik, 2020; SoftBank Robotics, 2020), as the following sections show. Mainly, reported solutions vary from the design of new robots, the adaption of existing devices for different purposes, to the implementation of commercial robotic platforms. Overall, the primary goal of those groups consists of providing efficient tools, exploiting the advantages of applying robotics or technology in the context of the COVID-19 pandemic (Brohi et al., 2020). This work focuses on three main categories (see Figure 1), which mainly seek to avoid propagating the virus, support the clinical staff, and ensure clean areas for both clinicians and patients.

### 2.1. Disinfection and Cleaning (DIS) Robots

Considering the high level of COVID-19 spread risk, the development and deployment of robots for clinical environment disinfection and decontamination have increased lately. The leading causes of infection in these areas include aspects as prolonged periods of exposure (unavoidable for clinical staff), conventional methods for cleaning (i.e., ineffective chemical inputs and human error), survivance of pathogens for long periods, and transmission through the hands between coworkers (Kramer et al., 2006; Otter et al., 2014; Boyce, 2016).

In this way, the inclusion of systems based on no-touch automated room disinfection (NTD) seeks to reduce the infection risk, removing human error, improving the effectiveness of the cleaning, and optimizing the disinfection times (Otter et al., 2013; Boyce, 2016; Marra et al., 2018). Robotic solutions of both commercial industries and research groups take strength in this period employing two cleaning principles: (1) spraying of chemicals and (2) ultraviolet (UV) light (Otter et al., 2013; Marra et al., 2018).

In the commercial context, specific-designed robots for disinfection and decontamination can be found, such as UVD Robot (UVD Robots, Odense, Denmark), Indoor Disinfection RoboCop (Milagrow Robotics, Gurgaon, India), SEIT-UV (Milvus Robotics, Ankara, Turkey), Anscer UVDR ALPHA (Anscer, Bangalore, India), ARIS-K2 (YOUIBOT, Shenzhen, China), CONNOR UVC Disinfection Robot (RobotLAB, San Francisco, USA), WDR01A (Wellwit Robotics, Shenzhen, China), LightStrike Germ-Zapping (Xenex, San Antonio, USA), Glosair 400 (Glosair, Champigny-sur-Marne, France), or Bioquell ProteQ (Bioquell, Andover, UK).

Robotic solutions have also been developed in scientific institutions, based on previous developments of mobile platforms and coupling disinfection mechanism as spraying or radiation. Moreover, although the interest in implementing those solutions has recently increased (e.g., XDBOT developed by the Nanyang Technological University), this field had previously presented significant advances in robotic systems (Andersen et al., 2006; Couto et al., 2017; Kovach et al., 2017; Yang et al., 2019).

For the Latin American autonomous robots, e.g., EXO-Andes UV-72 (EXO, Buenos Aires, Argentina), UVR-bot (UVRobotics, Buenos Aires, Argentina), Robot-UV (NFM Robotics, Callao, Peru), LD OMRON (Asahi, Aguascalientes, Mexico), RSD (Gesedic, Ciudad de Mexico, Mexico), and Thalon UV (Millenium BPO, Bogotá, Colombia) are also supporting the disinfection process. Furthermore, research projects in the region are providing robotic systems like the device developed by the Institute of Physics of Sao Carlos at the University of São Paulo for air decontamination.

### 2.2. Assistance, Service, and Logistics (ASL) Robots

Scenarios focused on assisting the clinical staff implies the use of robotics profiting characteristics as weight support capacity, smart navigation for mobile platforms, and precision in task execution, to name a few (Cremer et al., 2016). Recently, the development of robots for this application has not been very popular within the scientific community, resulting in the use of industrial platforms or social robots to support those tasks (Bloss, 2011).

Among the robotic tools that support the clinical staff in hospital environments for assistive applications, the following examples are found: Techi Buter (Techmetics Robotics, Santa Clara, USA), MiR100 (Mobile Industrial Robots, Odense, Denmark), TUG robot (Aethon, Pittsburgh, USA), RB-1 (Robotnik, Valencia, Spain), and Robotino (Festo, Esslingen am Neckar, Germany). Moreover, several studies have reported the use of these robots for logistic tasks, such as medication and food delivery (Kirschling et al., 2009; Takahashi et al., 2010, 2012; Bloss, 2011; Sermeus et al., 2016), patients transportation (Hu et al., 2011), medical equipment transportation (Wang et al., 2009; Ilias et al., 2014), environmental monitoring (Cremer et al., 2016; Mahdy et al., 2018), among others.

Regarding the Latin American context, few robotic applications for ASL have been reported. In Colombia, a food delivery startup is using a fleet of Kiwibot mobile robots (Kiwibot, Medellín, Colombia) to provide contactless deliveries (Meisenzahl, 2020). Although these robots are not being used in hospital environments, they can be easily adapted for healthcare support, as they are already equipped with sensing technologies for semi-autonomous navigation (Meisenzahl, 2020). Likewise, multipurpose robots are being implemented in this environment, such as RSD (Gesedic, Ciudad de Mexico, Mexico), whose mobile platform can be used in disinfection and assistance applications.

In the pre-pandemic period, the primary motivations consisted of delegating irrelevant activities to the robots to optimize the clinicians' time in patients' attention and executing heavy-weight tasks. Nevertheless, reducing the direct interaction of people with positive cases is also an attractive characteristic to implement this technology nowadays.

### 2.3. Telemedicine and Telepresence (TEL) Robots

Telemedicine is a general concept that encompasses any medical activity involving an element of distance (Wootton, 2001). Thus, this concept uses technology to provide a wide variety of clinical services through robots, the Internet, wireless devices, satellite, and telephone media (Achenbach, 2020). The expected benefits of telemedicine are mainly related to the faster access to health professionals, leading to optimization and improvement of the clinician's attention capacity (Hjelm, 2005; Achenbach, 2020). However, telemedicine takes hold in this pandemic time, changing a previously known drawback in its most significant advantage: the social distancing.

Different social robots are being adapted and applied to the telemedicine concept with significant growth in this period (Khan et al., 2020). Renowned platforms, such as Pepper (SoftBank Robotics, Tokyo, Japan) heads the list of robots to be called to keep the patient-clinician communication, even without representing a contagion risk (Podpora et al., 2020). This task is not unknown by the platform, since several studies have shown the potential of Pepper working in this application (Pandey and Gelin, 2018; Stock and Merkle, 2018).

In general terms, the common factor in the use of telemedicine robots involves mobile platforms integrated with videoconferencing hardware, such as RP-7 (Petelin et al., 2007; Rincon et al., 2012; Bettinelli et al., 2015; Garingo et al., 2016), RP-Vita (Sucher et al., 2011), Telepresence (Dao et al., 2019), Robotino (Tonin et al., 2011; Dobrev et al., 2018), BESSY (Murray et al., 2014). Similarly, humanoid-type robots are also found within this category, such as Stevie (McGinn et al., 2020), XR-1 (CloudMinds, Cayman Islands), Roy the robot (Smith et al., 2005), SCITOS (Hebesberger et al., 2017), among others.

Furthermore, Latin American initiatives, e.g., RED (Gesedic, Ciudad de Mexico, Mexico), RoomieBot COVID-19 (Roomie IT Services, Ciudad de Mexico, Mexico), as well as collaborative projects like EVA (PwC—RoboticsLab, Chile), have high potential and they are already being used in Telemedicine applications in this pandemic time. Similarly, a higher education institution of the Colombian government has developed a mobile robot to assist isolated patients due to COVID-19 (SENA, 2020). The robot of the National Learning Service (SENA) allows temperature taking and videoconferencing with family members and health professionals (SENA, 2020).

## 3. Materials and Methods

According to the above, this work presents the design and implementation of a perception questionnaire to assess healthcare providers' level of acceptance and education toward robotic solutions for the COVID-19 pandemic. In particular, several questionnaires were proposed to evaluate the perception of medical robotics, as well as of three types of robotics platforms for COVID-19 mitigation and control: (DIS) Disinfection and cleaning robots, (ASL) Assistance, Service, and Logistic robots; and (TEL) Telemedicine and Telepresence robots. This section describes the designed questionnaires and the experimental protocol.

### 3.1. Perception Assessment

A qualitative survey-based study was designed to assess health professionals' concepts, ideas, perceptions, and attitudes toward robotics in the management of the COVID-19 pandemic. The proposed surveys and questions are described below.

#### 3.1.1. Knowledge, Attitude, and Practice (KAP) Questionnaire

A quantitative questionnaire was developed to gather information on what health professionals know, how they feel and how they behave about disinfection (DIS), assistance (ASL), and telemedicine (TEL) robotic tools. In this sense, this study was based on the formulation of questions about the knowledge, attitudes, and practices of health care professionals regarding robotic tools for COVID-19 pandemic management and control. The first part of the survey was designed using knowledge-oriented questions. These questions measure the level of awareness and understanding that healthcare professionals have regarding robotic tools for DIS, ASL, and TEL. The second part was designed using attitude-oriented questions. These questions measure how healthcare professionals feel about robotic tools for DIS, ASL, and TEL, as well as any preconceived ideas or beliefs they may have about this topic. The third part was designed using practice-oriented questions. These questions provide insight into how healthcare professionals apply their knowledge and attitudes regarding robotic tools for DIS, ASL and TEL through their everyday actions.

Table 2 describes the proposed questions for the Knowledge, Attitude, and Practice (KAP) survey. Remarkably, *yes* or *no* questions were rated using 1 and −1 scores, respectively. Regarding the questions asking to rate experience or knowledge about a topic, a 5-point Likert scale was used, which were then converted to a scale from −2 to 2 points. Finally, questions formulated as statements were also evaluated using 5-point likert scales, and then they were converted to a scale from −2 to 2. Table 2 also illustrates the minimum and maximum score for each type of question.

**Table 2:** Designed questions for the Knowledge, Attitude, Perception (KAP) survey used in this study.

**Category**	**Question**	**Type of robot**	**Minimum score**	**Maximum score**
K	Have you ever heard of medical robotics?	ROB	−1	1
	Have you ever seen a health care robot?	ROB	−1	1
	Have you ever interacted with a health care robot?	ROB	−1	1
	Did you know about cleaning and disinfection robots?	DIS	−1	1
	Rate your experience with robots for cleaning and disinfection.	DIS	−2	2
	Rate your knowledge about the benefits of cleaning and disinfecting robots.	DIS	−2	2
	Did you know the robots for assistance and logistics?	ASL	−1	1
	Rate your experience with robots for assistance and logistics.	ASL	−2	2
	Did you know the robots for telemedicine?	TEL	−1	1
	Rate your experience with telemedicine robots	TEL	−2	2
	Rate your knowledge about the benefits of robots for telemedicine.	TEL	−2	2
A	In general, robots are useful.	ROB	−2	2
	I consider robots to be useful in medicine and health care.	ROB	−2	2
	I think robots in medicine and health care could replace people.	ROB	2	−2
	I think robots in medicine and health care improve service delivery.	ROB	−2	2
	I believe that disinfection and cleaning robots can mitigate and control the effects of the COVID-19 pandemic.	DIS	−2	2
	I believe that robotic assistance and logistics in hospital settings can mitigate and control the effects of the COVID-19 pandemic.	ASL	−2	2
	I believe that telemedicine robots can mitigate and control the effects of the COVID-19 pandemic.	TEL	−2	2
P	How often do you discuss about health robots in your work?	ROB	−2	2
	How often do you use or interact with robots for disinfection and cleaning?	DIS	−2	2
	I would recommend robotic tools for disinfecting and cleaning in my work.	DIS	−2	2
	How often do you use or interact with robots for assistance and logistics?	ASL	−2	2
	I would recommend robotic tools for assistance and logistics in my work.	ASL	−2	2
	How often do you use or interact with robots for telemedicine?	TEL	−2	2
	I would recommend robotic tools for telemedicine in my work	TEL	−2	2

*ROB stands for questions oriented to assess robotics in general. DIS stands for questions oriented to assess disinfection and cleaning robots. ASL stands for questions oriented to assistance and logistics robots. TEL stands for questions oriented to telemedicine and telepresence robots*.

#### 3.1.2. Perception Toward Robotics for COVID-19 in Colombia

To assess healthcare professionals' perceptions of the possibilities and scope of robotics for pandemic management in Colombia, an additional short questionnaire was proposed. The purpose of this questionnaire was to determine whether participants considered Colombia to have potential capabilities to develop robotic solutions for disinfection, care and telemedicine, or whether there are barriers to the development of these platforms.

Table 3 describes the proposed questions. These questions were formulated as statements and participants were asked to respond at what level they agreed with them, using a 5-point Likert scale. These questions were then converted to a scale from −2 to 2.

**Table 3:** Proposed questions to assess the perception of healthcare providers toward medical robotics for COVID-19 in Colombia.

**Tag**	**Question**	**Minimum score**	**Maximum score**
QCOL1	I believe that Colombia can develop robots to mitigate and control the effects of the COVID-19 pandemic	−2	2
QCOL2	In the case of Colombia, I believe that there are barriers to implement the robots that are in the international market	2	−2
QCOL3	I believe that robotic tools for disinfection, assistance and telemedicine should be acquired in Colombia	−2	2

#### 3.1.3. Open Questions

Finally, three additional open questions were designed to identify the functionalities that clinicians consider useful and necessary in DIS, ASL, and TEL robots. Table 4 describes the proposed questions.

**Table 4:** Proposed open questions to identify key functionalities of disinfection, assistance, and telemedicine robots.

**Tag**	**Question**
QO1	Briefly describe the features that you think a cleaning and disinfection robot should have.
QO2	Briefly describe the features that you think a robot should have for assistance and logistics.
QO3	Briefly describe the features that you think a telemedicine robot should have.

### 3.2. Experimental Protocol

This section describes the designed experimental procedure to apply the questionnaires for perception assessment in a group of healthcare professionals. Similarly, this section summarizes the session environment and the demographic information of the volunteers who took part in this study.

#### 3.2.1. Session Environment

This study was carried out in two private healthcare institutions in the city of Bogotá D.C., Colombia. The two clinics were selected because they have been treating patients with COVID-19 since the beginning of the pandemic. Additionally, selecting the clinics for this study also required professionals working in intensive care units.

#### 3.2.2. Session Procedure

Participants were asked to virtually fill out the perception questionnaires, using the Google Forms online tool. At the beginning of the form, participants were presented with the informed consent, which they had to read carefully and accept before proceeding with the form. Afterward, participants were asked for demographic information about their profession and their work environment. Preceding the questionnaires, a brief description of each type of robot was presented (i.e., DIS, ASL, and TEL), to homogenize the definition of such devices among the participants. Table 5 describes the definitions that were used with the participants. Moreover, the questionnaire also included the visual description presented in Figure 1. Finally, the questionnaires were applied.

**Table 5:** Brief description of the different robot categories that were used in the study.

**Tag**	**Category name**	**Description provided to participants**
TEL	Telemedicine and telepresence robots	During this survey, robots for telemedicine will be understood as those robots that allow accompanying patients who are in isolation, monitoring patient's vital signs remotely, and performing patrol and awareness tasks. This category does not include surgical robots.
DIS	Cleaning and disinfection robots	During this survey, cleaning and disinfection robots will be understood as those devices that allow the decontamination, sterilization and elimination of pathogens in different environments. Generally, these robots use ultraviolet light technology, chemical spraying systems or cleaning systems with disinfectant substances.
ASL	Assistance and logistics robots	During this survey, robots for assistance and logistics will be understood as those devices that allow the distribution of medicines in an automated way, automated catering or food distribution, sample and/or waste management, delivery of medical instruments and patient management.

*This information was provided to the participants prior to the fulfillment of the questionnaires*.

#### 3.2.3. Participants Recruitment

Before the recruitment of volunteers, this study was approved by the Escuela Colombiana de Ingeniería Julio Garavito ethics committee. The subjects were all formally recruited to participate in this study voluntarily, and provided their signed consent form. The informed consent clarified that participants would not have any repercussions on their job because of the responses collected. Moreover, the data was stored without any identifier to determine the source of the answers.

The inclusion criteria were as follows: adults over 18 years old, healthcare professionals working in hospital environments can read and sign the informed consent form. The exclusion criteria were as follows: subjects with declared conflicts of interest with this study.

From the two clinics, 41 healthcare professionals voluntarily participated in this study, who were contacted by email. This sample size follows the criteria reported in previous studies that involve the use of surveys Vasileiou et al. (2018). Table 6 summarizes the demographic information of the subjects. In particular, 20 women and 21 men with an average age of 35.39 ± 8.48 years were involved in the study. Approximately 83% of the participants indicated a work experience of more than 2 years. Additionally, 43.9% of participants indicated that they work in intensive care units or surgery, and 70% of participants responded that their daily work activities implied contact with COVID-19 patients.

**Table 6:** Demographic data of the healthcare personnel who participated in the study.

**Participants**	**41**	
**Gender**	20 female	21 male
**Age**, mean (SD)	35.39 (8.48) years	
**Healthcare profession**		
- Physiatrist	4.87%	
- Physical therapist	14.64%	
- Occupational therapist	9.75%	
- Biomedical engineer	9.75%	
- Health technologist	4.87%	
- Nursing auxiliary	7.31%	
- Surgical instrumentalist	4.87%	
- Anesthesiologist	19.57%	
- Respiratory therapist	19.57%	
- Nurse/Medical intensivist	4.86%	
**Experience**		
−0–2 years	17.07%	
−3–5 years	24.39%	
−6–7 years	24.39%	
−8–10 years	9.75%	
- Over 11 years	24.39%	
**Educational level**		
- Bachelor's degree	48.78%	
- Master's degree	2.43%	
- Post-graduate studies	36.58%	
- Technologist	12.19%	
**Healthcare working area**		
- Rehabilitation	21.95%	
- Hospitalization	9.75%	
- Imageology	4.87%	
- Surgery	21.95%	
- Sterilization	4.87%	
- Intensive care	21.95%	
- NA	14.65%	

### 3.3. Data Analysis

All data was virtually collected and then processed using Microsoft Excel and R Studio software. In relation to the KAP questionnaire, quantitative indicators related to the scores obtained by each participant were estimated. To determine if there were differences between participants with positive and negative knowledge levels about robotics, all scores were separated and compared between these two conditions. To assess the existence of significant differences the non-parametric Mann-Whitney *U* test was used. This test was selected, considering that it has been reported to have minimal type I error rates, as well as, equivalent power with *t*-test for Likert scales (Joost and Dodou, 2010). Likewise, for small sample sizes this test presents better results than *t*-test (Blair and Higgins, 1980).

## 4. Results

A total of 41 surveys were satisfactorily fulfilled, with all participants completing the proposed form. No survey was discarded and all participants reported that the questions were clear and understandable. As a further result, none of the participants reported being infected with SARS-CoV-2. The data of this results are available in a public repository at https://doi.org/10.6084/m9.figshare.13373741. This section describes and illustrates the primary outcomes of this study.

### 4.1. Knowledge, Attitude, and Practice (KAP) Questionnaire

**Figure 2:**
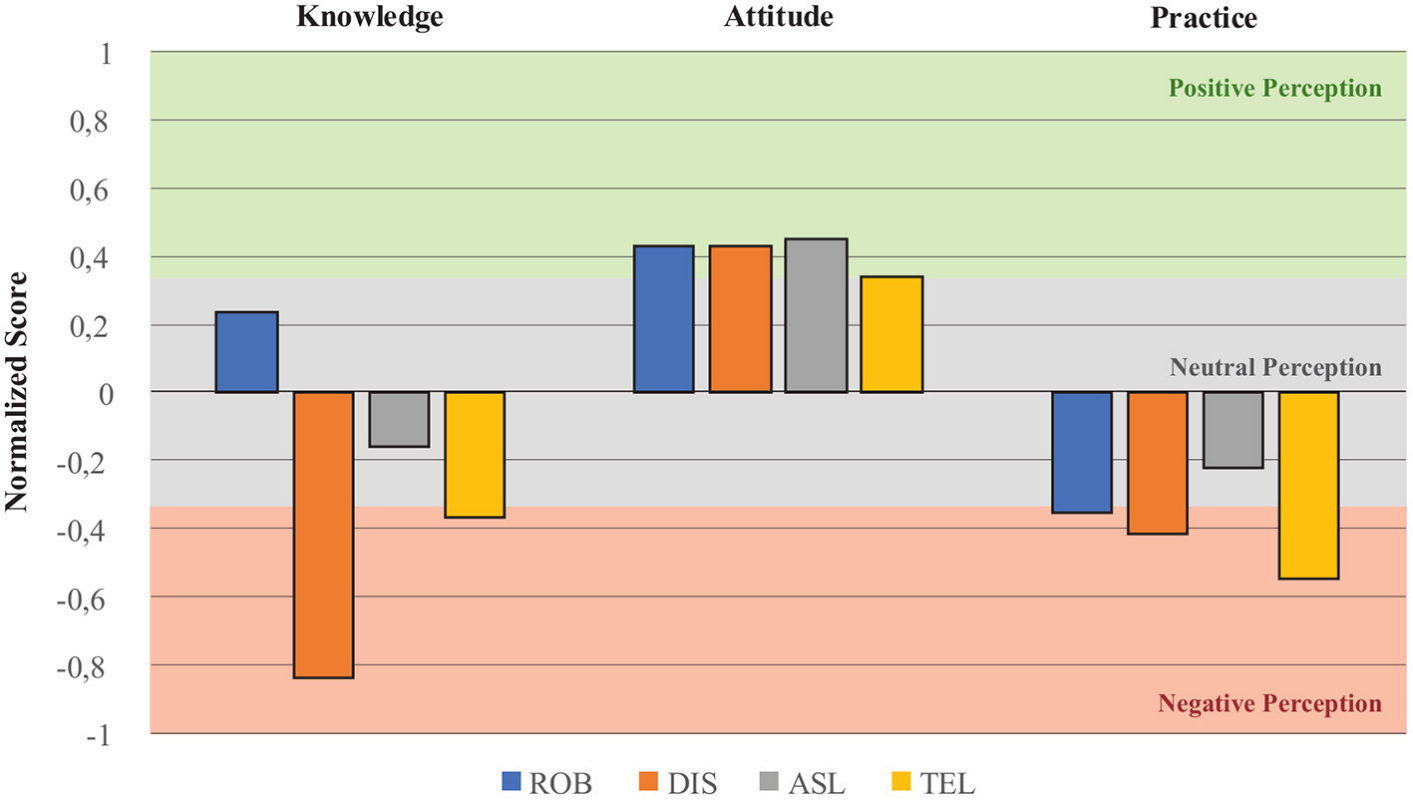
Results of the Knowledge, Attitude, and Practice (KAP) survey. The scores for each type of robot were grouped and normalized to identify the overall perception. ROB stands for questions oriented to medical robotics. DIS stands for questions oriented to disinfection robots. ASL stands for questions oriented to assistance, service, and logistics robots. TEL stands for telemedicine and telepresence robots. The data standardization used the possible maximum and minimum values of each question, being inverted the values for questions formulated negatively.

Regarding the KAP survey, Figure 2 summarizes the primary outcomes of the proposed questions. For each type of robot (i.e., ROB, DIS, ASL, and TEL), the questions were grouped into three categories: knowledge-oriented questions, attitude-oriented questions, and practice-oriented questions. Moreover, considering the maximum and minimum scores described in Table 2, the scores obtained for questions of the same category and type of robot were averaged for each participant. The data was normalized through the maximum and minimum values that can be obtained in each question, being inverted for questions formulated negatively. Finally, an overall normalized score was obtained by averaging the scores of all the participants. In Figure 2, the normalized scores are displayed between −1 and 1, indicating a negative to positive perception scale. For analysis purposes, such an scale was equally divided into three zones, namely negative, neutral, and positive perception.

Moreover, to assess if there was a difference in perceptions between those participants who reported a negative knowledge about robotics and those who reported a positive one, a comparison of the average scores obtained with the KAP survey was performed between these conditions. Table 7 illustrates the comparison between the scores for all participants, the scores for participants with negative knowledge, and the scores for participants with positive knowledge about robotics. Furthermore, to determine the existence of significant differences between these conditions, the non-parametric Mann-Whitney *U* test was performed. Thus, Table 7 also describes the obtained *p*-values with this test.

**Table 7:** Comparison of average scores obtained for the participants with a negative knowledge about robotics (ROB) and the participants with a positive knowledge about robotics (ROB).

**Category**	**Type of robot**	**Scores**			**Negative vs. Positive *p*-value**
		**All (*n* = 41)**	**Negative knowledge (*n* = 16)**	**Positive knowledge (*n* = 25)**	
K	ROB	0.24	−0.38	0.63	**0.00001**
	DIS	−0.84	−0.75	−0.90	**0.04136**
	ASL	−0.16	−0.34	−0.05	**0.02144**
	TEL	−0.37	−0.53	−0.26	0.20766
A	ROB	0.43	0.40	0.45	0.55520
	DIS	0.43	0.50	0.38	0.38430
	ASL	0.45	0.41	0.48	0.62414
	TEL	0.34	0.34	0.34	0.96012
P	ROB	−0.35	−0.81	−0.06	**0.00022**
	DIS	−0.41	−0.28	−0.50	**0.05486**
	ASL	−0.22	−0.39	−0.11	**0.01140**
	TEL	−0.55	−0.63	−0.50	**0.03078**

*P-values in bold indicate significant differences (p < 0.05) between participants with negative and positive knowledge*.

### 4.2. Robotics for COVID-19 in Colombia

**Figure 3:**
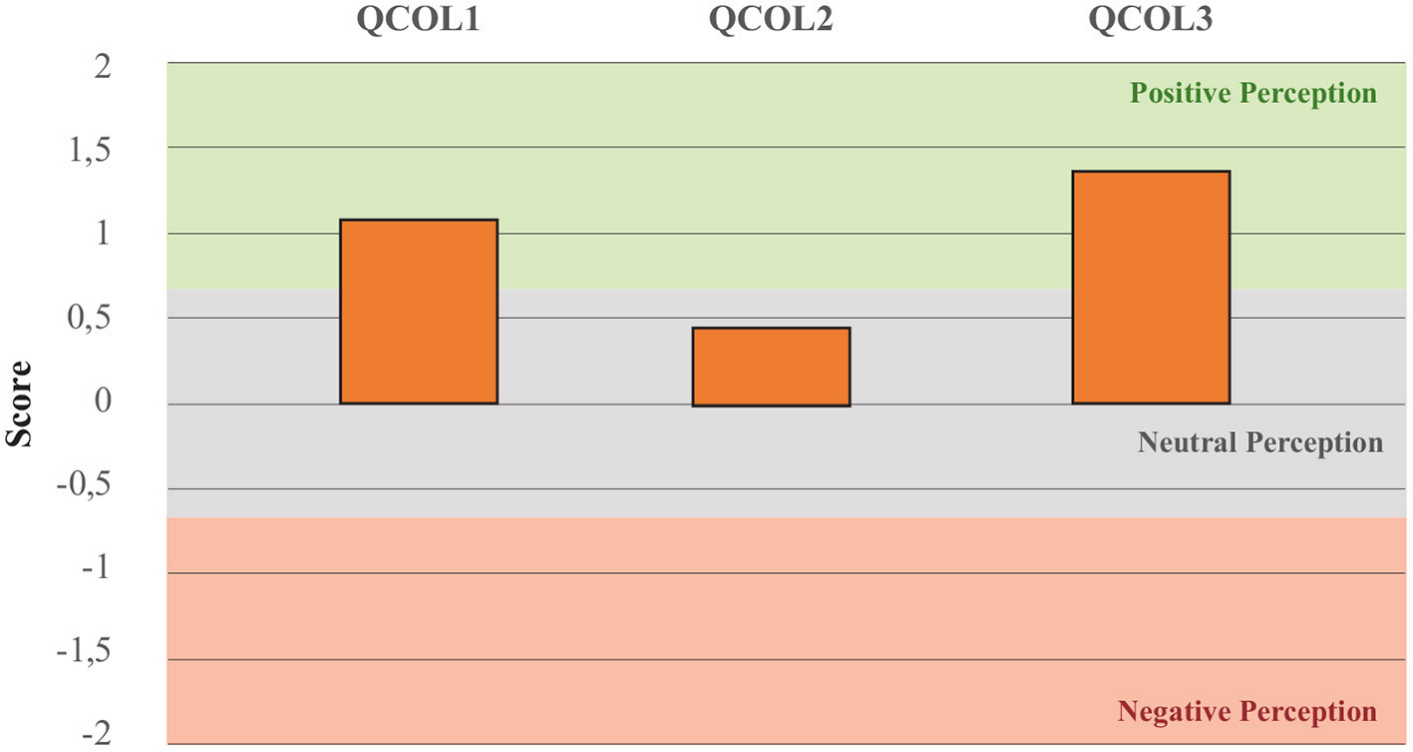
Results of the perception questions toward the scope of Robotics for COVID-19 in Colombia. QCOL1 assess the perception of the potential to develop robotic solutions in Colombia. QCOL2 assess the perception of barriers to implement robotic solutions. QCOL3 assess the need for robotic solutions for disinfection, assistance and telemedicine during COVID-19 pandemic.

To identify the perceptions of the healthcare professionals about the scope of medical robotics during COVID-19 in Colombia, the questions presented in Table 3 were applied. Figure 3 summarizes the average score for each question.

### 4.3. Open Questions

**Figure 4:**
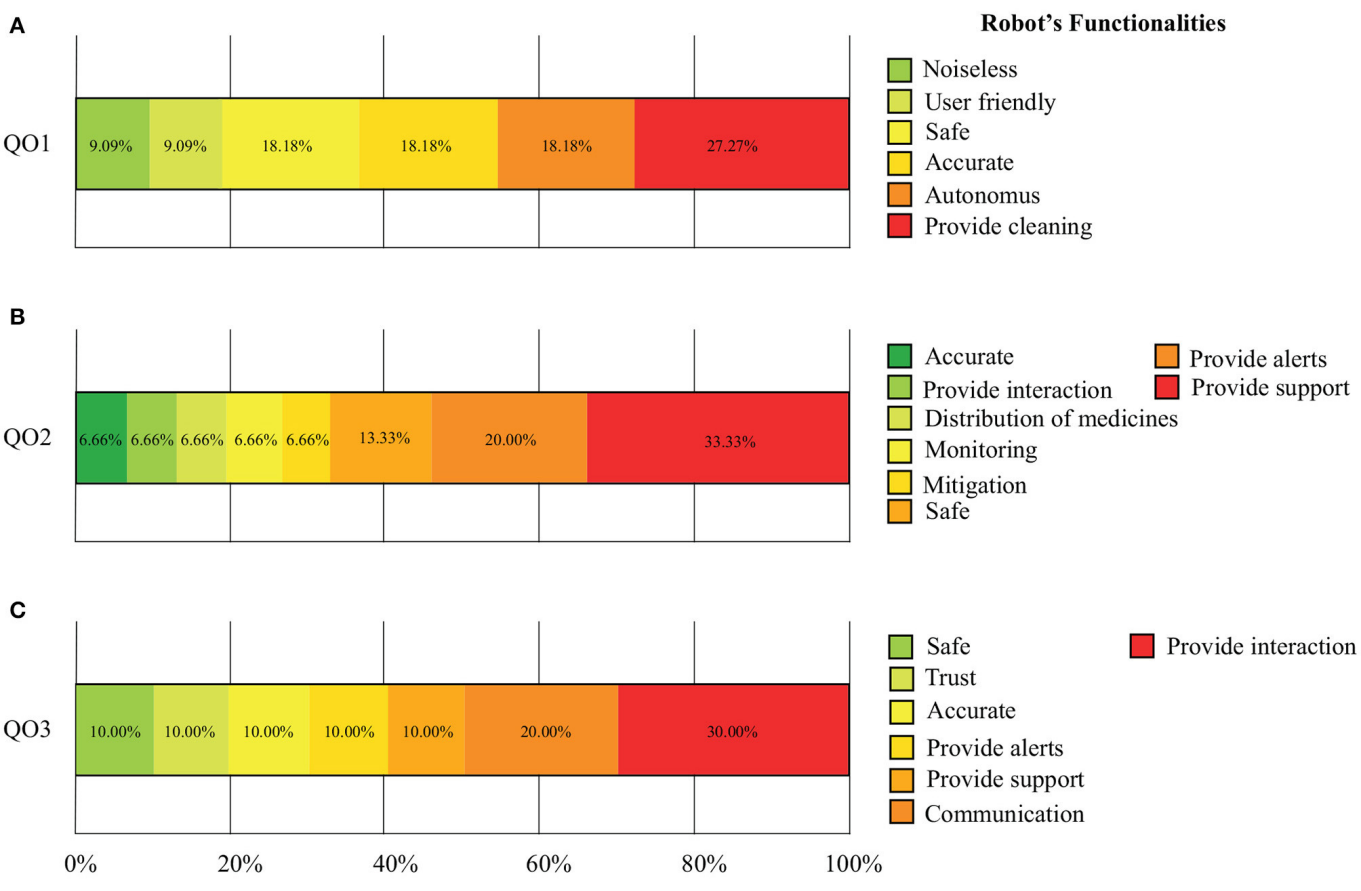
Results of the proposed open questions to identify key functionalities of disinfection, assistance, and telemedicine robots. **(A)** Disinfection and Cleaning Robots. **(B)** Assistance, Service, and Logistics Robots. **(C)** Telemedicine and Telepresence Robots.

Finally, Figure 4 presents the results of the open questions proposed in Table 4.

## 5. Discussion

All the subjects successfully completed the online questionnaires, and no cases of misunderstanding were reported. The outcomes from the KAP survey, the Colombian context, and the open questions are discussed as follows.

### 5.1. Knowledge, Attitude, and Practice (KAP) Questionnaire

Regarding the KAP survey, several outcomes related to the three constructs of the questionnaire can be assessed (i.e., knowledge, attitude and practice). First, it can be established that there is a positive level of knowledge about medical robotics in general for the surveyed population. However, concerning robots for disinfection (DIS), assistance (ASL), and telemdicine (TEL), participants indicated that they have a low level of knowledge and experience with these types of robots. This result may imply that although professionals recognize medical robotics as a potential tool to assist their work, they do not have sufficient awareness or education about robots' functions and features for DIS, ASL, and TEL.

Conversely, although the level of awareness was low, participants reported a positive attitude toward robots' usefulness and benefits in managing and controlling the COVID-19 pandemic. In particular, one of the attitude-oriented questions sought to determine whether health professionals believed robotics could replace them. If participants responded that they agreed with the statement, it was considered to be a negative attitude. In this case, 60.9% of the participants answered “neither agree nor disagree,” and only 29.3% responded that they disagreed with the statement. This result may imply that it is necessary to carry out education and awareness processes in the medical community (Goh and Sandars, 2020), to strengthen the idea that robots can enhance and improve their work, but they cannot replace the healthcare professionals fundamental activities. For instance, Coombs (2020) recommends performing a familiarization stage based on culture theory to understand individuals' social practices when interacting with the technology and their preferences within its usages. This culture theory will increase their motivation and trust toward technology, such as medical robotics. Additionally, following design methods, such as Design Thinking and Design Sprint can be useful to create user-friendly applications, and more acceptable devices within the medical community (White et al., 2020).

Regarding practice-oriented questions, the common denominator among the participants' answers indicates that healthcare professionals do not frequently use nor interact with robots in their work. An interesting result was obtained regarding whether participants would recommend using robots for DIS, ASL and TEL in their work. In particular, for disinfection (DIS) robots, only 19.5% of the participants agreed to recommend them in their work. For assistance, service, and logistics (ASL) robots, 65.8% of participants agreed to recommend them in their work. However, for telemedicine (TEL) robots, 48.8% of participants did not agree to recommend them in their work. These outcomes follow the ideas highlighted by some researches in the use of robotics mostly to assist the patients through platforms that could navigate in hostile clinical environments (Yang et al., 2020), and perform medical delivery tasks (Feil-Seifer et al., 2020). Furthermore, to reduce the reluctance to DIS and TEL robotics platforms is essential to include healthcare personnel in training programs to elucidate robots' importance and capabilities during the pandemic. Also, a challenge could be DIS and TEL comprehensive tools that can integrate features of ASL platforms.

Finally, to evaluate if there were differences in the perceptions of participants with positive (61%) and negative (39%) knowledge about robotics, the results of the KAP survey were separated and compared accordingly. For analysis purposes, the participants that reported positive knowledge will be referred to as the *positive group*, and the participants that reported negative knowledge will be referred to as the *negative group*. As presented in Table 7, comparing the knowledge scores (K) for disinfection (DIS) and assistance (ASL) robots, significant differences were found between negative and positive groups. Regarding DIS robots, Although the positive group scored more negatively than the whole group, this result may be explained by the fact that health professionals, who have notions of robotics, have commonly worked with or seen robots related to surgery, rather than robots associated with disinfection tasks. Conversely, regarding ASL robots, the positive group reported a neutral knowledge about them, whilst the negative group reported more negative scores than the whole group, as expected.

In relation to the attitude questions, no significant differences were found between the positive and negative groups for any type of robot. This result can be explained because in spite of the level of knowledge and conscientiousness of the health professionals, their attitude remains positive, as they recognize the robotics' usefulness and benefits in hospital environments.

In relation to the practice questions, significant differences were found for all types of robots between the positive and negative groups. For robotics in general (ROB), the positive group reported neutral scores, similar to the whole group. However, the negative group reported very negative scores, indicating that owing to the little knowledge about robotics, robots are not commonly used in their daily activities. Regarding DIS robots, the positive group reported negative scores, while the negative group reported neutral scores. This result suggests that regardless of knowledge about robotics in general, the level of awareness and in healthcare professionals about the benefits of robots for disinfection (DIS) is still low. With regards to ASL robots, the positive group exhibited a neutral distribution, similar to the whole group. In contrast, the negative group consequently reported the absence of practices and use of ASL robots in their daily tasks. Lastly, both the positive and negative groups reported poor practices related to TEL robots; however the scores from the negative group were slightly more negative.

### 5.2. Robotics for COVID-19 in Colombia

With regards to the perception of the participants toward the capacities and needs for robotics amid the COVID-19 in Colombia, several aspects were identified. First, question QCOL1 was aimed at determining if the participants considered that Colombia has enough technological advances to develop robotic solutions for the COVID-19 pandemic. In particular, 82.9% of participants indicated that they agreed that Colombia could develop such robotic solutions. Second, question QCOL2 was intended to determine if the participants considered barriers to the deployment of robotic platforms available in the international market. In this case, a slightly positive perception was obtained, where 40% of the participants indicated that they disagreed that there were barriers to the implementation of robots from the international market for the COVID-19 pandemic. Finally, question QCOL3 sought to identify if participants considered that robotic tools for DIS, ASL and TEL should be acquired in Colombia. In this case, 87.8% of participants agreed with this statement.

There are few publications related to robotic-tools for the COVID-19 pandemic in Colombia. Some of the applications, propose the use of robotic arms to sustain physical distancing between patients and doctors (Guerra et al., 2020), disinfection robots to support clinical neurophysiology studies (San-Juan et al., 2020), and teleoperation robots to monitor patients and connect doctors (Forbes Staff, 2020). Thus, the opportunities for developing robotics tools in Colombia during and after the pandemic are increasing to answer the healthcare sector needs.

### 5.3. Open Questions

Finally, this study also sought to provide insights into the features that robots should have for COVID-19 management, according to the opinions of healthcare professionals. Particularly, question QO1 was focused on identifying the expectations regarding the functionalities of disinfection robots. As it can be seen, the healthcare personnel answered that the robot must provide cleaning tasks, be safe and accurate. In a lower percentage, the clinicians recommended that the device has to be noiseless and user friendly. On the other hand, Question QCOL2 was intended to assess the clinicians' expectations regarding assistance and logistics robots. The healthcare personnel highlighted the importance of these robots, to support clinicians' tasks, and to trigger alerts as advice for emergency or important events. Finally, Question QCOL3 was focused on evaluating the clinician's opinions regarding the telemedicine and telepresence robots for the COVID-19 pandemic. The outcomes showed that the healthcare staff expects that these robots can socially interact within hospital environments, and communicate with users, connecting patients and doctors.

Overall, the healthcare personnel seeks for safe and accurate robotic systems. Therefore, the efforts of deploying robotics for COVID-19 have to be focused on optimizing and building tools with high precision, and increase safety strategies (Otter et al., 2013; Marra et al., 2018). Similarly, the work by (Tavakoli et al., 2020) remarked the features that robotics should have (i.e., autonomy, monitor, provide support and interaction) to collaborate in healthcare scenarios, not only to manage the adverse effects of the COVID-19 pandemic but also to support prevention processes.

### 5.4. Final Remarks

In this work, the sample size might be considered as small, however it follows the criteria reported in previous studies that involve the use of surveys (Vasileiou et al., 2018). Moreover, although the participants were only recruited from two healthcare institutions in Bogotá D.C., Colombia, this is the first study that describes the perceptions and expectations of healthcare professionals toward robotics for COVID-19 in Colombia. Particularly, several KAP surveys on COVID-19 have been reported in literature; however, they were aimed at assessing the overall perception toward COVID-19 in patients and survivors, and they did not evaluate robotics perception for COVID-19 outbreak management (Ferdous et al., 2020; IFRC Turkish Red Crescent, 2020; REACH, 2020).

## 6. Conclusions

This paper presented clinician's perception toward DIS, ASL, and TEL robots amidst the COVID-19 pandemic. A total of 41 participants completed an online KAP (i.e., Knowledge, Attitudes, and Perception) survey, as well as two short questionnaires about medical robotics.

In general, the outcomes showed that participants have a positive level of knowledge regarding medical robots in general. However, the clinicians' experience and knowledge regarding DIS, ASL, and TEL platforms are shallow. Consequently, their awareness and education have to be increased in order to understand the opportunities, functions, and features of these tools. Furthermore, as reported in the literature, a familiarization stage in the first instance is recommendable to increase healthcare personnel's trust and motivation. This stage will achieve the successful adaptation of the technology during the COVID-19 pandemic and after the outbreak.

Despite this level of awareness, participants elucidate a positive attitude toward robots in managing and mitigating the effects of the COVID-19 pandemic. In particular, 65.8% of clinicians recommend using ASL robots in the pandemic, which remark the clinicians' preferences for platforms capable of supporting logistic tasks, medication and food delivery, and monitoring the environment. In the case of DIS and TEL platforms a lower perception was presented. Hence, the efforts concerning these technologies have to be in increase the clinicians' trust and develop comprehensive platforms capable of providing assistance and disinfection or teleoperation.

Additionally, a very encouraging result is the healthcare positive perception regarding the capabilities in Colombia to develop these tools. Although few studies propose the development of robotic platforms to assist medical procedures in Colombia, the opportunity to increase the research and advances regarding DIS, ASL, and TEL robots are very high.

Regarding the robot's functionalities. The participants highlight the importance of building safe and accurate systems in general. For DIS robots, the healthcare staff's primary characteristic is that the robot provides reliable cleaning and autonomy. In the ASL robots case, the significant features were to provide support and provide alerts to attend emergency events. Finally, for TEL the results suggest that the main capabilities are to provide interaction and communication. Concluding, these results demonstrate that DIS, ASL and TEL platforms hold the promising potential to be a feasible approach to support COVID-19 pandemic from different approaches. One last interesting result of this work is related to the fear in health professionals to be replaced by robots. In particular, the participants' opinions were not very conclusive since ~60% of the participants assumed a neutral position when asked if they considered that they could be replaced. However, when relating the findings of the functionalities that robots should provide to improve health service, participants agreed that robots should perform repetitive and non-critical tasks, such as transporting medications and cleaning. In this sense, it can be stated that “being replaced” by a robot does not necessarily imply a negative perception if robots assist in less essential tasks.

Future works will address the validation and implementation of this survey in multiple Latin American countries to provide a more deep comparison and assessment of healthcare providers' perception. Moreover, future studies will also be focused on identifying the specific opinions of healthcare professionals toward existing DIS, ASL, and TEL robotic platforms in both national and international markets.

## Data Availability Statement

The datasets presented in this study can be found online at: https://doi.org/10.6084/m9.figshare.13373741.

## Ethics Statement

The studies involving human participants were reviewed and approved by the ethics committee at the Colombian School of Engineering Julio Garavito. The patients/participants provided their written informed consent to participate in this study.

## Author Contributions

DG-V, FR, PB, SR, MB, and RC performed the literature review. SS, DG-V, MM, and CC designed the methodology. SS and DG-V conducted the experimental sessions. SS and NC performed the data curation and processing. SS, DG-V, and NC wrote the original manuscript. MM, FR, PB, SR, MB, RC, and CC reviewed and edited the manuscript. MM and CC supervised the study and managed the funding resources. All authors contributed to the conceptualization of the study.

## Conflict of Interest

The authors declare that the research was conducted in the absence of any commercial or financial relationships that could be construed as a potential conflict of interest.
